# circ_0084927 promotes cervical carcinogenesis by sponging miR-1179 that suppresses CDK2, a cell cycle-related gene

**DOI:** 10.1186/s12935-020-01417-2

**Published:** 2020-07-21

**Authors:** Xinhua Qu, Liumei Zhu, Linlin Song, Shaohua Liu

**Affiliations:** 1 Department of Obstetrics, Yantai Affiliated Hospital, Binzhou Medical College, No. 717 Jinbu Street, Muping District, Yantai, 264100 Shandong China; 2 Department of Maternal and Child Health Promotion, Yantai Affiliated Hospital, Binzhou Medical College, No. 717 Jinbu Street, Muping District, Yantai, 264100 Shandong China

**Keywords:** Cervical cancer, circ_0084927, miR-1179, CDK2

## Abstract

**Background:**

Cervical cancer (CC) is a malignant tumor found in the lowermost part of the womb. Evolving studies on CC have reported that circRNA plays a crucial role in CC progression. In this study, we investigated the main function of a novel circRNA, circ_0084927, and its regulatory network in CC development.

**Methods:**

qRT-PCR was applied to evaluate the expression of circ_0084927, miR-1179, and CDK2 mRNA in CC tissues and cells. Dual-luciferase reporting experiments and RNA immunoprecipitation (RIP) assay were conducted to validate the target relationship of miR-1179 with circ_0084927 and CDK2 mRNA. CCK-8 and BrdU assays were also used to evaluate CC cell proliferation. The adhesion and apoptosis phenotypes of CC cells were measured using cell–matrix adhesion and caspase 3 activation assay. Flow cytometry was also employed to detect the CC cell cycle.

**Results:**

Our results indicated that circ_0084927 was up-regulated in CC tissues and cells. Findings also revealed that circ_0084927 silence inhibited CC cell proliferation and adhesion while facilitating apoptosis and triggering cell cycle arrest. However, miR-1179 down-regulation appeared in CC tissues. Apart from observing that circ_0084927 abolished miR-1179’s inhibitory effects on cell proliferation and adhesion, it was found that CDK2 was up-regulated in CC tissues and was instrumental in cancer promotion. Also observed was that miR-1179 directly targeted CDK2, thereby inhibiting CDK2’s promotion on the malignant phenotypes of CC cells. Lastly, results indicated that circ_0084927 revoked the inhibitory effect of miR-1179 on CDK2 by sponging miR-1179.

**Conclusion:**

circ_0084927 promoted cervical carcinogenesis by sequestering miR-1179, which directly targeted CDK2. Our results also provided novel candidate targets for CC treatment in that it revealed the circ_0084927/miR-1179/CDK2 regulatory network that strengthened CC aggressiveness.

## Background

Cervical cancer (CC) is a malignant tumor that seriously threatens women’s life. The estimated incidence rate of CC is 13.1 per 100,000 women, with Africa and China accounting for a third of the global incidence [1]. Human papillomavirus (HPV) infection has been considered to be the main cause of CC, and preventive vaccines have been developed not only to suppress CC occurrence but also to prevent HPV infection [1–3]. Although the HPV vaccine, screening and prevention help reduce the infection rate of CC, a high incidence of CC persists in society [4, 5]. Surgical treatments combined with chemotherapy or radiotherapy have improved the survival outcome of CC patients, yet the treatment effect in high-risk patients has remained poor [6–8]. Immunotherapy studies have also provided a new potential treatment of CC [9]. Moreover, the mortality rate of patients with CC has continued to soar because CC is prone to metastasis and recurrence [10]. For this reason, the exploration of new early treatment and diagnosis methods for CC is of extraordinary significance in saving the lives of women with CC.

Circular RNAs (circRNAs) refer to a class of non-coding RNAs with covalently linked ring structures, which prevent them from being degraded by exonucleases [11]. These RNAs perform a variety of biological functions, such as regulating genes. In recent years, a growing number of studies have shown that circRNAs are inextricably linked to the malignant process of many cancers [12–16]. Some researchers confirmed that circRNAs could promote CC [17–19], while some suggested that multiple circRNAs were associated with the pathological process of CC [20–22]. One study reported that the up-regulation of certain circRNAs appeared in CC tissues [23]. More interesting is that reconstructive analysis of CC indicated a complicated circRNA‑miRNA‑mRNA regulatory network [24]. Even though research on the underlying molecular mechanisms of circRNAs in CC has attracted a great deal of attention from scholars, the literature is yet to investigate whether circ_0084927 plays a regulatory role in CC. This knowledge vacuum, therefore, explains the importance of investigating the role of circ_0084927 as well as its potential regulatory network in CC.

Regarded as a class of small endogenous non-coding RNAs commonly found in plants and animals, microRNAs (miRNAs) can interact with the 3′UTR of a target gene through complementary base pairing. Recent studies have shown that miRNAs act as a tumor promoter or tumor suppressor in the occurrence and development of CC [25–28]. For instance, different pieces of research confirmed that miR-1179 played an inhibitory role in several cancers, including glioblastoma, gastric cancer and non-small-cell lung cancer [29–31]. Besides, studies on miR-1179 showed that the abnormal down-regulation of miR-1179 accelerated the malignant process of breast cancer and pancreatic cancer [32, 33]. Nonetheless, scientists are yet to clarify whether miR-1179 exerts a role in CC regulation.

Cyclin-dependent kinase 2 (CDK2) gene, located on the 12q13.2 chromosome, consists of eight exons. As a member of the protein kinase family, CDK2 participates in the regulation of the eukaryotic cell division cycle [34]. Apart from the fact that a previous study uncovered that CDK2-related signaling pathways conferred complicated roles in several forms of cancers [35], a few studies have investigated the tumor-promoting role of CDK2 in CC [36–38]. In another research, bioinformatics analysis authenticated that the CDK2-related signaling pathway was involved in CC [39]. Experimental results also suggested that CDK2 was a downstream target of some miRNAs in CC progression [40, 41]. Despite these findings, no researcher has investigated whether CDK2 could be regulated by miR-1179 in CC. The interactome involving CDK2 and circRNA also remains unclear.

This study aimed to explore the role of circ_0084927 in CC development and reveal how the molecular mechanisms and regulatory networks of circ_0084927 affect CC. Our results showed that circ_0084927 promoted CC occurrence by sequestering the inhibitory effect of miR-1179 on CDK2.

## Materials and methods

### Sample acquisition and cell culture

CC tissue samples were collected from Yantai Affiliated Hospital of Binzhou Medical College, China, and the study protocols were approved by the Ethics Committee of Yantai Affiliated Hospital of Binzhou Medical College. CC cell lines (HeLa, CaSki, SW756 and C-33A) and normal cervical epithelial cell lines (HcerEpic) were purchased from the Beijing Beina Chuanglian Biotechnology Research Institute (BNBIO.com). HeLa, C-33A, SW756, and CaSki cells were cultured in 5% CO_2_ at 37 °C in RPMI-1640 medium (E600028; Sangon, Shanghai, China) with 10% fetal bovine serum (16140071; Gibco; Thermo Fisher Scientific, Inc., Waltham, MA, USA) in addition to 100 μg/ml streptomycin. HcerEpic cells were cultured in 5% CO_2_ at 37 °C in MEM medium (E600024; Sangon, Shanghai, China) with 10% fetal bovine serum and 100 μg/ml streptomycin. The characteristics of the patients are shown in Table 1, and the representative histopathological examination results are illustrated in Additional file 1: Figure S1.

**Table 1:** The clinical characteristics of patients with cervical cancer

Characteristics	Case (30)
Age (years)	
≤ 55	13 (43.3%)
> 55	17 (56.7%)
FIGO stage	
I–IIa	20 (66.6%)
IIb, III–IV	10 (33.3%)
Tumor size	
≤ 4 cm	18 (60.0%)
> 4 cm	12 (40.0%)
Lymph node	
Negative	18 (60%)
Positive	12 (40%)

*FIGO* International Federation of Gynecology and Obstetrics

### H&E staining

Tissue sections were deparaffinized twice using xylene treatment (10 min each time), and they were re-hydrated by decreasing the alcohol concentration. After washing the tissue sections in distilled water for 1 h, they were stained by hematoxylin solution for 8 min and by eosin for 3 min. After that, the tissue sections were dipped in 0.2% saturated lithium carbonate solution for 30 s. The eosin solution was then used to stain the tissue sections for 1 min after washing the sections in running tap water. Finally, the H&E staining images were photographed with the Nikon TE2000-U inverted microscope (Japan).

### Cell transfection

The small interfering RNAs of circ_0084927 (si-circ_0084927) and CDK2 (si-CDK2), as well as the negative control siRNA (si-NC), were synthesized by GenePharma (Shanghai, China). Some items were purchased from RiboBio Co., Ltd. (Guangzhou, China), such as miR-1179 control, miR-1179 negative control, miR-1179 mimic (for luciferase reporter gene assay) and miR-1179 inhibitor. HeLa and C-33A cells were transfected with si-NC, miR-1179 inhibitor, si-circ_0084927, si-CDK2, miR-1179 inhibitor plus si-circ_0084927 or miR-1179 inhibitor plus si-CDK2 via Lipofectamine ™ 2000 (11668019; Thermo Fisher Scientific, Inc., Waltham, MA, USA) and via lipofectamine transfection method for 20 min. After the cells were incubated for 2 days at 37 °C, they were analyzed by qRT-PCR.

### Subcellular location using a nuclei-cytoplasm fractionation method

Before the nuclear and cytoplasmic RNA isolation, nuclear and cytoplasmic fractions were separated using the PARIS Kit (AM1921; Thermo Fisher Scientific, Waltham, Mass., USA). The isolated RNA products in nuclei and cytoplasm were analyzed by qRT-PCR. Then, the expression of circ_0084927 and ESRP1 mRNA was detected in the nuclei and cytoplasm. GAPDH and U2 were subsequently employed as a reference control for cytoplasmic expression and nuclear expression, respectively.

### qRT-PCR

The trizol reagents (15596026; Thermo Fisher Scientific, Inc., Waltham, MA, USA) were first used, according to the instruction manual, to isolate and detect total RNA from the tissue samples and cell lines. The obtained RNA was then reverse-transcribed into cDNA. Then, miR-1179 was reverse-transcribed using the protocol of mirVana™ qRT-PCR miRNA Detection Kit (AM1558; Invitrogen™; Thermo Fisher Scientific, Inc., Waltham, MA, USA). The reverse-transcription of CDK2 mRNA and circ_0084927 was conducted with SuperScript III First-Strand Synthesis SuperMix for qRT-PCR (11752050; Thermo Fisher Scientific, Inc., Waltham, MA, USA). StepOnePlus Real-Time PCR System (4376600; Thermo Fisher Scientific, Inc., Waltham, MA, USA) was later used to perform qRT-PCR. The qPCR products were then validated using the agarose gel electrophoresis method. Next, the data were analyzed with the 2^−ΔΔCt^ method. GAPDH was then utilized as the internal control of circ_0084927 and CDK2 mRNA, while U6 was used as the internal control of miR-1179. The primer sequences are displayed in Table 2.

**Table 2:** The primer sequences for RT-qPCR

Name	Primer sequences (5′–3′)
circ_0084927	
Forward	CGAAGGAACGGAGAAGCTCT
Reverse	GTGCCCTGACTACGGTGTTA
circ_0084912	
Forward	CTTGATGACCCCAGAAGGAG
Reverse	ATATTCCAGGCTTCCCAACC
circ_0081723	
Forward	CCATCACCGACCTCATCAGT
Reverse	TGATGTTTCCCAGTGTGTGG
circ_0106385	
Forward	GAGGAGGAGGAGAAGAATGC
Reverse	ACGTGGCACAGACCTCTCTC
circ_0099591	
Forward	CCAACCAATGAGTCGAAGGT
Reverse	CTCGGAGTGTGAGGGATAGC
miR-1179	
Forward	GCGCGCAAGCATTCTTTCAT
Reverse	GTCGTATCCAGTGCAGGGTCCGAGGTATTCGCACTGGTACGAACCAACCA
U6	
Forward	CTCGCTTCGGCAGCACA
Reverse	AACGCTTCACGAATTTGCGT
CDK2	
Forward	CCAGGAGTTACTTCTATGCCTGA
Reverse	TTCATCCAGGGGAGGTACAAC
β-Actin	
Forward	CATGTACGTTGCTATCCAGGC
Reverse	CTCCTTAATGTCACGCACGAT

### Luciferase reporter gene assay

Oligonucleotides comprising the circ_0084927 mutant (the sequence containing the miR-1179 binding site was mutated to GAUACGA) or the CDK2 mRNA 3′UTR mutant (the sequence containing the miR-1179 binding site was mutated to GAUACGA) were synthesized by GenePharma (Shanghai, China). Inserted into the dual-luciferase miRNA target expression vector (pGL4) were wild-type circ_0084927, circ_0084927 mutant, CDK2 3′UTR mutant, and wild-type CDK2 3′UTR. This insertion was performed to construct luciferase reporter plasmid. HeLa and C-33A cells were also co-transfected with luciferase porter plasmid and miR-1179 mimic. After 48 h of incubation, the culture medium was removed to collect the cells. The collected cells were then lysed to obtain cell lysates. The luciferase activity was measured by Pierce Renilla-Firefly Luciferase Dual Assay Kit (16185; Thermo Fisher Scientific, Inc., Waltham, MA, USA) according to the protocol.

### RNA immunoprecipitation (RIP) assay

The Hela and C-33A cells transfected with miR-1179 mimic were cultured to an appropriate density. The cultured cells were digested using trypsin and were collected after trypsin treatment. The cells were lysed using RIP lysis buffer. The cell lysates were then incubated with RIP buffer containing magnetic beads coupled with anti-Argonaute2 (MA5-23515; Thermo Fisher Scientific, Inc., Waltham, MA, USA) or IgG for 1 h, with IgG serving as a negative control. The mixture was subsequently incubated with Proteinase K. After that, the immunoprecipitated RNA was isolated and analyzed using qRT-PCR.

### RNA pull-down assay

RNA pull-down assay was conducted to further validate the regulatory binding relationship between miR-1179 and CDK2 mRNA. The biotinylated double-stranded RNA of miR-1179 (Bio-miR-1179) and biotinylated negative control RNA (Bio-NC) were designed by GenePharma (Shanghai, China). Whereas the sense sequence of bio-miR-1179 was 5′-AAGCAUUCUUUCAUUGGUUGG-biotin-3′, the antisense sequence of bio-miR-1179 was 5′-CCAACCAAUGAAAGAAUGCUU-3′. 1 × 10^5^ Hela and C-33A cells were cultured in 6-well plates for 1 day, resuspended in 1 ml lysis buffer, and incubated in ice for 20 min. The lysate was centrifuged at 12,000×*g* for 15 min before the supernatant was collected. The mixture of bio-miR-1179 or bio-NC and streptavidin-coated magnetic beads (Invitrogen, USA) was added to the supernatant and incubated at 4 °C for 2 h. The pulled-down CDK2 mRNA in the bio-miR-1179 or bio-NC group was detected by qRT-PCR.

### CCK-8 assay

After the transfected cells underwent trypsinization, 100 μl of the transfected cell suspension was seeded into a 96-well plate (2 × 10^3^ cells/well). The plate was then placed in a 37 °C incubator for some hours (24 h, 48 h, and 72 h). Then, 10 μl of CCK-8 solution was added to each well, based on the manual guidelines of CCK-8. After the cells were incubated with CCK-8 for 2 h, the absorbance was measured at 450 nm.

### BrdU incorporation ELISA assay (A colorimetric BrdU assay)

BrdU cell proliferation assay kit was used to detect cell-proliferation ability. Anti-BrdU antibodies were used to detect 5-bromo 2′-deoxyuridine (BrdU), which was incorporated into the cell DNA during cell proliferation. Then, a trypsin-treated suspension containing 10^4^ cells was added to each well of a 24-well plate. The culture medium was changed every 6 h. After the cells were cultured for 24 h, 10 μm BrdU (E607203; Sangon, Shanghai, China) was added, and the culturing was continued for 4 h to allow the proliferating cells to incorporate BrdU into their DNA. The cultured cells were then fixed using the fixing solution and were permeabilized with 0.5% Triton (R) X-100 for 10 min. Mouse anti-IgG and anti-BrdU antibodies (diluted at 1:50) were then incubated with the cells overnight at 4 °C. Subsequently, cells were washed with PBST and incubated in the dark with HRP-conjugated secondary antibodies (A24494; Thermo Fisher Scientific, Inc., Waltham, MA, USA) at 1:500 in PBS at room temperature. In the end, the absorbance at 450 nm was proportional to the amount of BrdU incorporated into the cell, which directly reflected cell proliferation.

### Cell–matrix adhesion assay

Before the cell suspension (10,000 cells/well) was seeded in the 96-well plates (4414133; Thermo Fisher Scientific, Inc., Waltham, MA, USA) that were previously coated with 10 μg/ml type I collagen (C7661, Sigma-Aldrich, USA), the cells were deprived of serum for at least 8 h. After 30 or 60 min adherence at 37 °C in a 5% CO_2_ atmosphere, the wells were washed with PBS for at least three times to remove the non-adherent cells. The remaining cells were then treated with MTT for two more hours at 37 °C. Finally, the MTT-treated cells were treated with 100 µl DMSO. The absorbance recorded using a microplate reader (Benchmark, Bio-Rad, USA) was 570 nm.

### Assays for caspase 3 activation

Caspase-3 is an active cell-apoptosis protease and an early indicator of the onset of apoptosis. Colorimetric detection at 405 nm of p-nitroaniline (pNA), after the cleavage from the peptide substrate DEVD-pNA, may reflect the cell apoptosis level. The transfected Hela and C-33A cells (1x10^5^) in different groups were briefly harvested and lysed in 50 ml of ice-cold cell lysis buffer. Cell lysates were centrifuged at 10,000*g* for 10 min to obtain the supernatant. Then, 50 μl of 2× Reaction Buffer/DTT Mix and 5 μl of 1 mM DEVD-pNA (substrate for caspase-3) from caspase 3 colorimetric assay kit (630217, Takara Biomedical Technology (Beijing) Co., Ltd., China) were added to the cell lysates. The absorbance was determined by measuring OD405 of the released pNA using a microplate reader (Benchmark, Bio-Rad, USA).

### Western blot analysis

Quantitative analysis was performed after all the protein was extracted with RIPA lysis buffer (C500005, Sangon; Shanghai, China) from Hela and C-33A cells in different groups. An equal amount of protein was separated by 10% SDS-PAGE. The gel was immersed in a transfer buffer to achieve equilibrium before transferring it to a polyvinylidene fluoride membrane. Primary antibodies were diluted at a ratio of 1:1000. The membrane was later incubated for 2 h with diluted primary antibodies against CDK2 (D220395; Rabbit-Human; Sangon; Shanghai, China) and β-actin (SAB5500001; Rabbit-Human; Sigma-Aldrich, China). Following that, the hybrid membrane was blocked with 5% skimmed milk and incubated at 4 °C overnight. Next, the membrane was incubated for 2 h with diluted secondary antibodies (A32731; Goat-Rabbit; Thermo Fisher Scientific, Inc., Waltham, MA, USA). Finally, a hypersensitive ECL chemiluminescence kit (C510043; Sangon; Shanghai, China) was used to detect proteins according to the reagent instructions. The intensity of the protein bands was read using ImageJ software.

### Cell cycle by flow cytometry

The transfected HeLa and C-33A cells were re-suspended once in pre-chilled 1xPBS and were subsequently diluted to 1 × 10^5^ cells/ml in 1× Annexin binding buffer. In every assay, 100 µl of cell suspension (10,000 cells) was used. The transfected HeLa and C-33A cells were then re-suspended and treated with pure ethanol for 30 min. After that, cells were incubated with RNase for 30 min not only to remove RNA but also to eliminate the influence of the binding between PI and RNA. Cells were subsequently stained with the red-fluorescent stain, PI (V13242; Thermo Fisher Scientific, Inc., Waltham, MA, USA), in a dark room at room temperature to allow PI to bind to the DNA of the cells. The stained cells were finally put into a flow cytometer before the proportion of cells in each phase of the cell cycle was obtained from the linked BD FACSuite software.

### Statistical analysis

With Microsoft Excel, all the means and standard deviations were calculated based on three independent experiments. GraphPad Prism 8.0 (GraphPad Prism, Inc., La Jolla, CA, USA) was used to produce the diagrams. One-factor analysis of variance (ANOVA) test and Student’s *t* test were used for the statistical analysis between multiple groups and for the statistical analysis of two groups, respectively. In terms of the gene expression in tissue samples, we used the Wilcoxon test for the CC tissue samples and matched adjacent healthy tissue samples for comparison. P < 0.05 was considered to be statistically significant, while P < 0.01 was considered to be extremely significant.

## Results

### circ_0084927 was selected as the circRNA of interest in CC

**Fig. 1:**
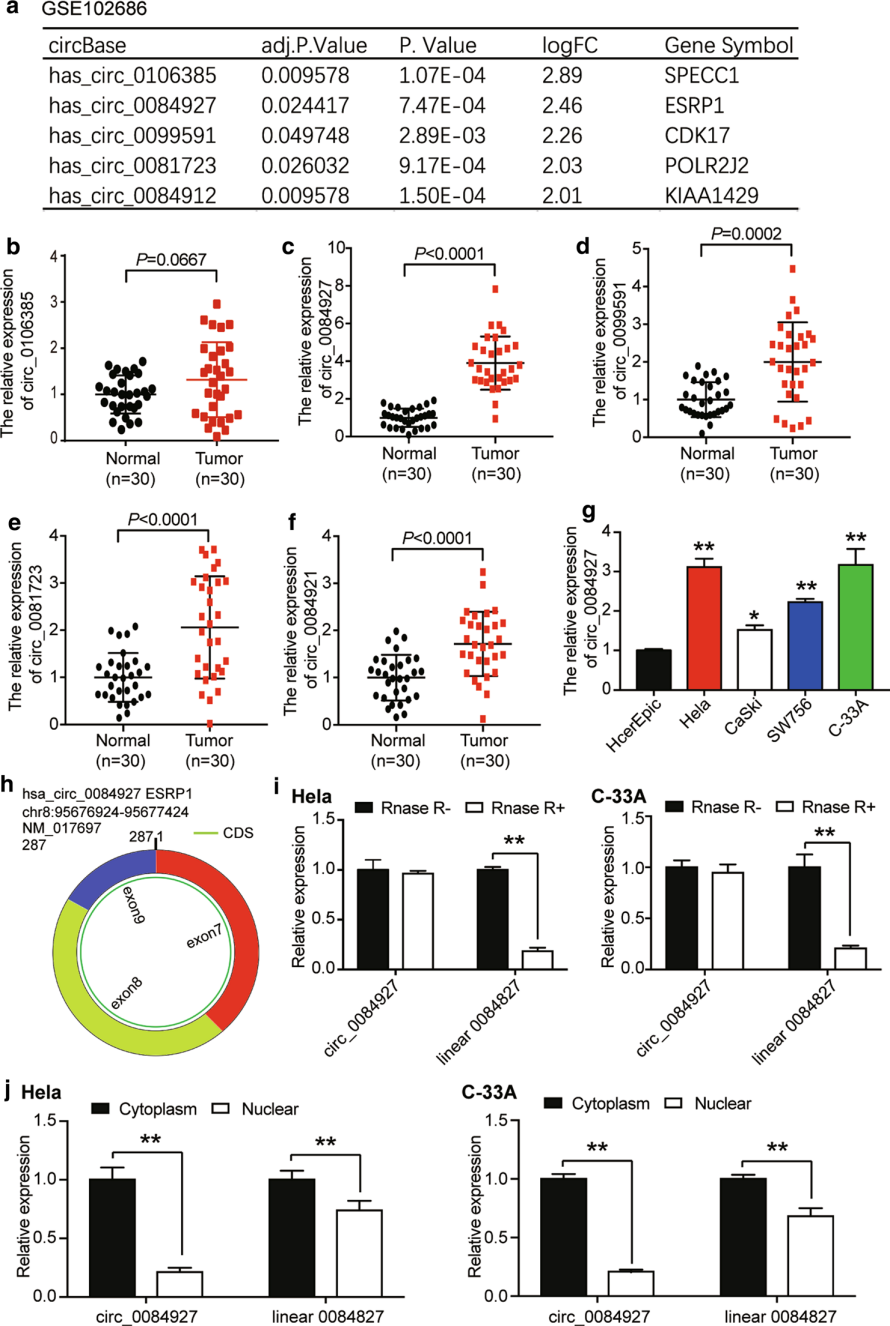
The identification and characteristics of circRNA. **a** By analyzing GSE102686 data series, we identified 21 differentially expressed circRNAs in cervical cancer, and the top five most significantly overexpressed circRNAs were presented here. The criteria for differential expression was adj. P < 0.05 and log|FC| ≥ 1.5. **b**–**f** The qRT-PCR results for the top five most significantly upregulated circRNAs in our collected tumorous cervical tissues and healthy adjacent tissues. N = 30. **g** qRT-PCR results for the expression of circ_0084927 in selected cell lines. Apart from the HcerEpic cell line, others are cervical cancer cell lines. *P < 0.05, **P < 0.01, compared with the HcerEpic cell line. **h** The structure of circ_0084927 was illustrated. It consists of three exons 7–9 from ESRP1 host gene. **i** The RNase R + tolerance feature of circ_0084927 in Hela and C-33A cell lines. **P < 0.01, compared with the control group without RNase R treatment. **j** The localization of circ_0084927 in Hela and C-33A cell lines using a cell fractionation method. **P < 0.01, compared with the level of circ_0084927 or linear ESRP1 mRNA in cytoplasm

We analyzed the GSE102686 data series using the GEO2R algorithm and identified 21 differentially expressed genes (DEGs). The top five most significantly up-regulated circRNAs included circ_0084927, circ_0106385, circ_0099591, circ_0081723, and circ_0084912 (Fig. 1a). The expression of these five circRNAs was evaluated in the obtained tissue samples. The results of qRT-PCR showed that excluding circ_0106385, circ_0084927, circ_0099591, circ_0081723, and circ_0084912 were significantly up-regulated in CC tissues than were in paired healthy cervical tissues (Fig. 1b–f). We then selected circ_0084927, which had the -highest expression level, as the research object. Further analysis of circ_0084927 expression revealed that compared to the normal cervical epithelial cell line (HcerEpic), circ_0084927 was significantly up-regulated in CC cell lines, including HeLa, CaSki, SW756, and C-33A. In particular, Hela and C-33A cell lines showed more than twofold circ_0084927 expression of the HcerEpic cell line (Fig. 1g). For this reason, they were selected for follow-up studies. It was observed that circ_0084927 was a closed circular RNA generated from and contained exons 7, 8 and 9 of its host gene, ESRP1 (Fig. 1h). To further characterize circ_0084927, we performed RNase R degradation experiments on HeLa and C-33A cells. Our analysis showed that RNase R greatly reduced linear_0084927 expression. Yet it had little effect on circ_0084927 expression in both cell lines (Fig. 1i). In addition, subcellular fractionation location analysis of HeLa and C-33A cells suggested that circ_0084927 and linear_0084927 were mainly located in the cytoplasm (Fig. 1j).

### miR-1179 was identified as a bridge effector of circ_0084927 and CDK2 in CC

Metascape.org was first employed to analyze the enriched terms of 904 DEGs (selection criteria: adj. P < 0.01 and log|FC| ≥ 2) from GSE63514 data series. As shown in the bar graph (Additional file 2: Figure S2A), the cell cycle was the most significantly enriched terms by the algorithm of Metascape.org. In particular, critical genes involved in the cell cycle-process of CC can be seen through an MCODE profile (Additional file 2: Figure S2B).

**Fig. 2:**
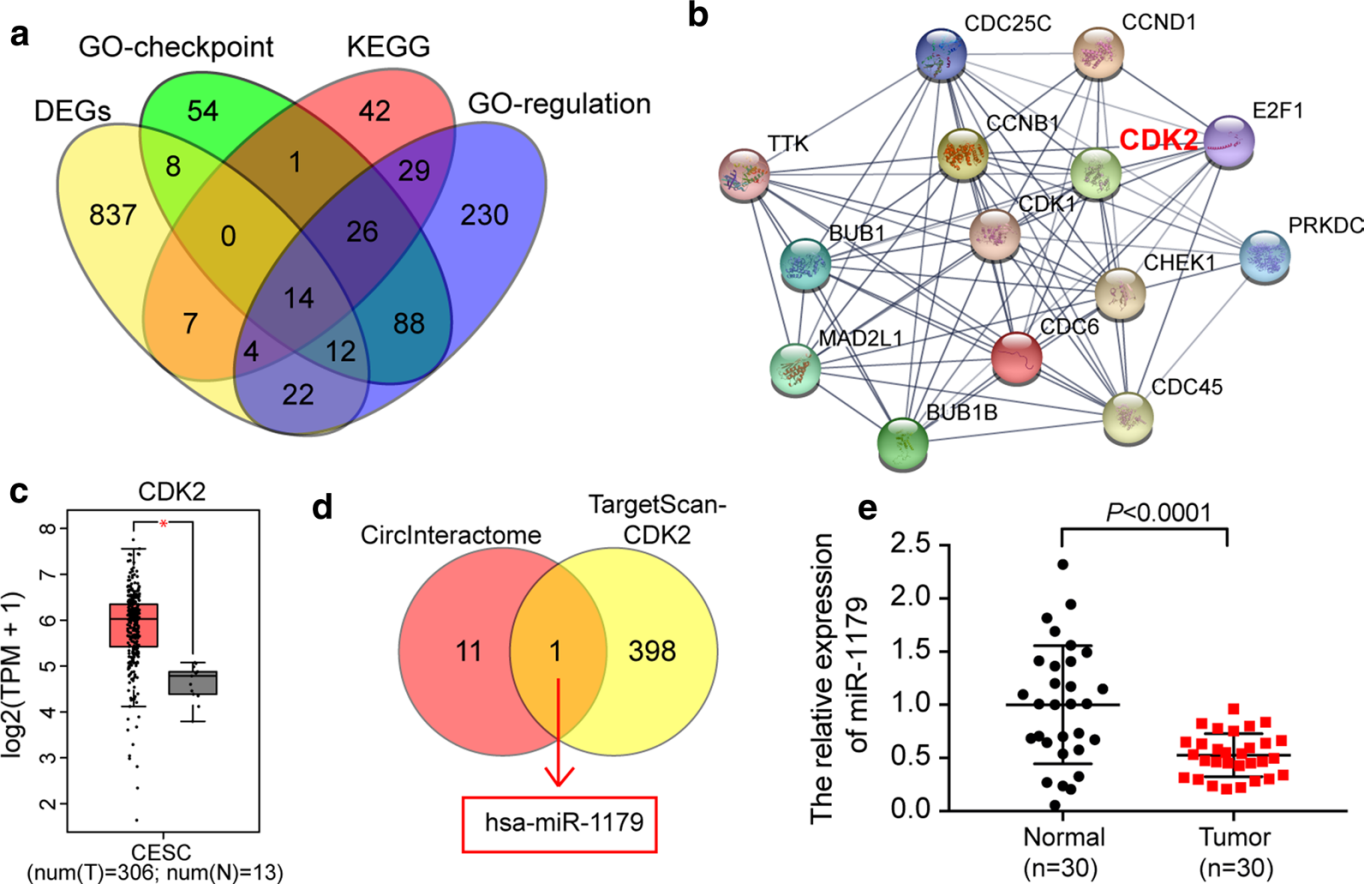
The identification of mRNA and miRNA. **a** The DEGs involved in the three GSEA datasets were screened out, and 14 of them went through STRING analysis (**b**). Among the genes in the STRING network, we noticed that CDK2 had been studied thoroughly in cervical cancer; however, its networking with circRNAs had been studied conclusively. Thus, CDK2 was chosen as our gene of interest in this study. **c** GEPIA algorithm produced the relative expression of CDK2 in cervical squamous cancer (CESC). Num = number. T = tumor, N = healthy normal. **d** The identification of miRNA can be sponged by circ_0084927 and target CDK2 mRNA. The downstream miRNAs of circ_0084927 was predicted by circular RNA interactome (https://circinteractome.nia.nih.gov/), while the upstream miRNAs of CDK2 mRNA were predicted by TargetScan Human 7.2. Finally, miR-1179 was identified. **e** The relative expression of miR-1179 in our collected tissue samples

After conducting a GSEA analysis of all genes in GSE63514, we found that cell cycle-related processes were all up-regulated in CC, such as the cell cycle, regulation of cell cycle phase transition, and cell cycle checkpoint process (Additional files 3, 4, 5: Figures S3, S4, S5). By intersecting the 904 DEGs of the GSE63514 data series, genes involved in the 3 cell cycle-related processes were identified by GSEA analysis. A total of 14 genes was identified (Fig. 2a). The 14 genes then went through STRING v11 (https://string-db.org/) protein–protein interaction network analysis. As shown in the network, CDK2 was significantly related to other proteins (Fig. 2b). Even though CDK2 had been studied thoroughly in cervical cancer, studies that focused on its networking with circRNAs were limited. Because of this, CDK2 was chosen as the gene of interest in this study.

By querying GEPIA expression data, we also found that CDK2 was significantly up-regulated in cervical squamous cancer (CESC) tissue samples (Fig. 2c). We then intersected downstream miRNAs of circ_0084927 predicted by circular RNA interactome (https://circinteractome.nia.nih.gov/), and the upstream miRNAs of CDK2 mRNA predicted by TargetScan Human 7.2 (http://www.targetscan.org/vert_72/). Finally, miR-1179 was identified (Fig. 2d). The relative expression of miR-1179 in our collected tissue samples was measured, and findings revealed that miR-1179 was significantly downregulated in CC tissues rather than in healthy control tissues (Fig. 2e).

### circ_0084927 directly repressed miR-1179 by targeted inhibition

**Fig. 3:**
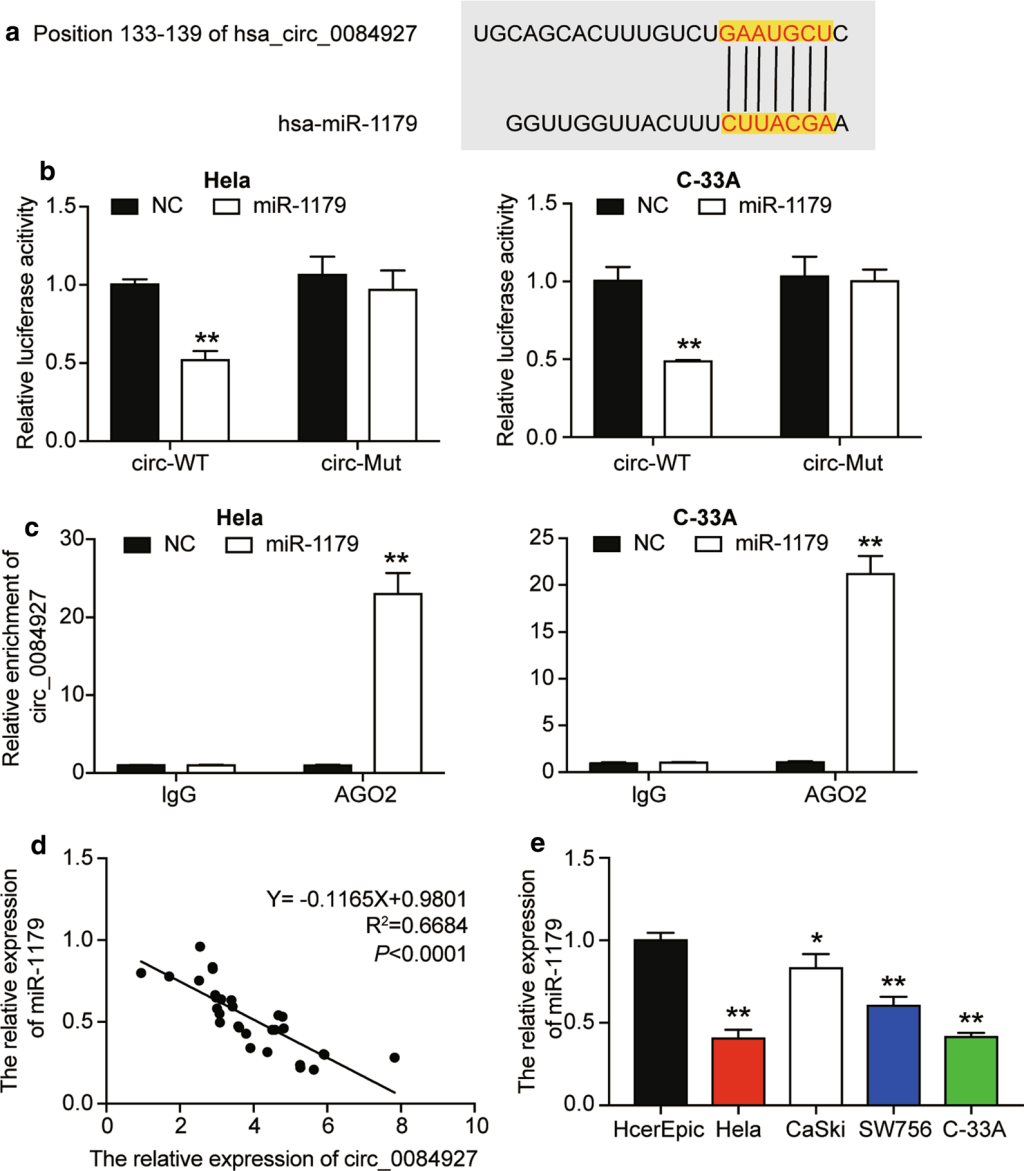
circ_0084927 directly targeted miR-1179. **a** The scheme showed that circ_0084927 interacted with the 3′UTR oligonucleotide sequence of miR-1179. The binding relationship was predicted by circular RNA interactome. **b** The potential binding between circ_0084927 and miR-1179 was validated by dual-luciferase reporter assay. circ_0084927 mutant or circ_0084927 wild-type plasmids containing fluorescein and miR-1179 mimic were co-transfected into HeLa cells and C-33A cells. **P < 0.01, compared with the NC group. **c** The interaction between circ_0084927 and miR-1179 was measured by RIP analysis. IgG was the negative control for AGO2, while NC was the negative control for miR-1179 mimic. **P < 0.01, compared with NC group. **d** The expression of miR-1179 in cervical cancer tissues was tested by qRT-PCR with U6 as an internal control. A negative correlation relationship between circ_0084927 and miR-1179 expression was identified by spearman correlation analysis. **e** The expression of miR-1179 in cell lines. *P < 0.05, **P < 0.01, compared with the HcerEpic cell line, which is the control cell line. **b**–**e** The data were in the form of mean ± SD of three experiments

In this study, circ_0084927 and miR-1179 were predicted to bind with each other via pairing in the GAAUGCU-CUUACGA manner (Fig. 3a). To verify the interaction of circ_0084927 and miR-1179, we mutated the GAAUGCU sequence of circ_0084927 that bound miR-1179 to GAUACGA. Luciferin-containing circ_0084927 mutant or circ_0084927 wild-type plasmids and miR-1179 mimic were co-transfected into HeLa and C-33A cells. It was found that the introduction of miR-1179 mimic into the cells attenuated the luciferase expression of circ_0084927 wild-type plasmids but that it had no effect on circ_0084927 mutant (Fig. 3b). RIP results also demonstrated that adding miR-1179 mimic precipitated circ_0084927 (Fig. 3c). However, miR-1179 expression displayed a negative correlation with circ_0084927 in CC tissues (Fig. 3d). Later, miR-1179 was discovered to be significantly downregulated in CC cell lines. Hela and C-33A cell lines showed approximately ½ lower miR-1179 levels than HcerEpic cell lines (Fig. 3e).

### circ_0084927 promoted cervical carcinogenesis by inhibiting miR-1179

**Fig. 4:**
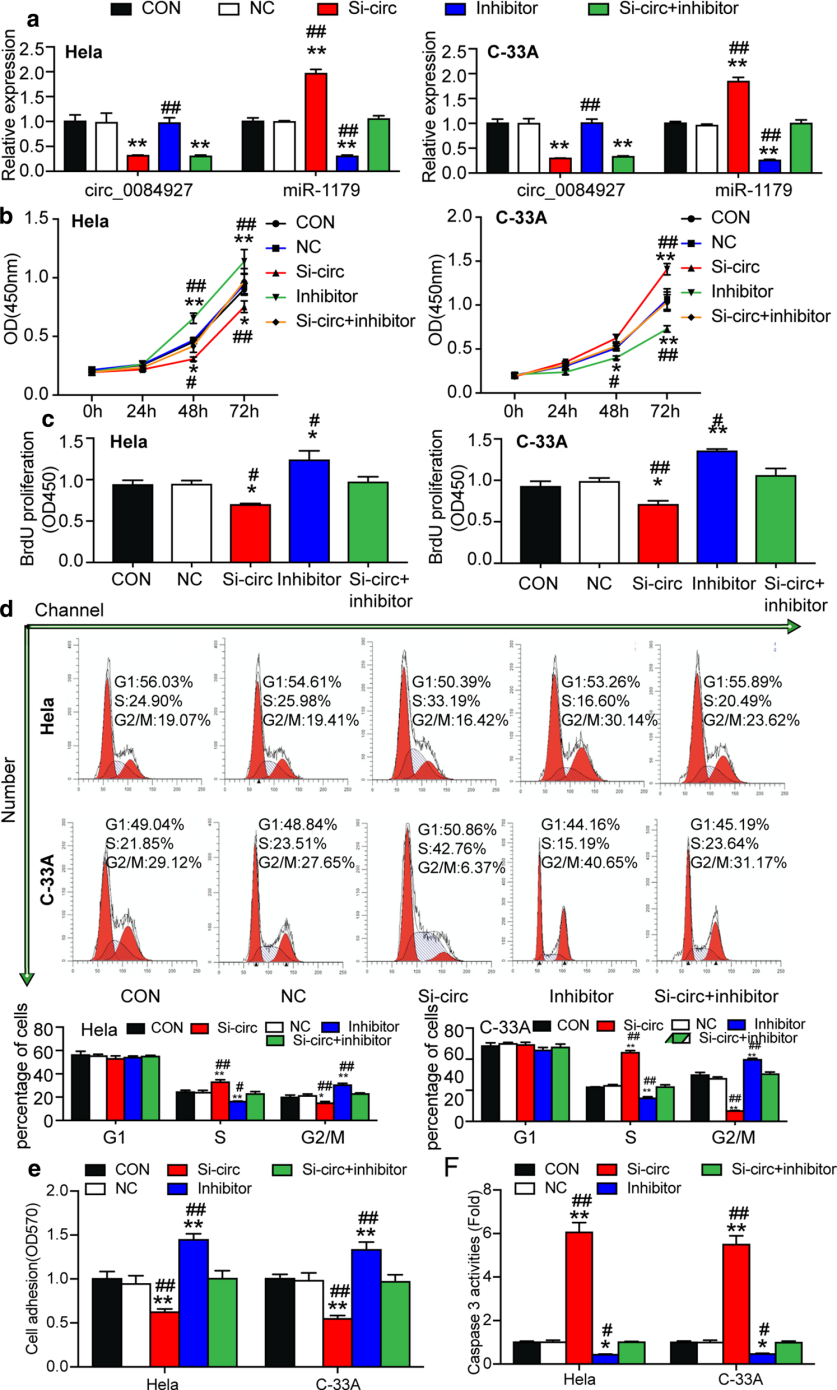
circ_0084927 enhanced cervical carcinogenesis by inhibiting miR-1179. **a** The transfection efficiency of si-circ_0084927 and miR-1179 inhibitor in transfected cells was detected by qRT-PCR. **b** The proliferation of HeLa and C-33A cells transfected with si-NC, si-circ_0084927, miR-1179 inhibitor or si-circ_0084927 plus miR-1179 inhibitor was determined by CCK-8 assay. **c** BrdU incorporation assay was used to analyze the proliferation of HeLa and C-33A cells transfected with si-NC, si-circ_0084927, miR-1179 inhibitor or si-circ_0084927 plus miR-1179 inhibitor. **d** The cell cycle progression of HeLa and C-33A cells transfected with si-NC, si-circ_0084927, miR-1179 inhibitor or si-circ_0084927 plus miR-1179 inhibitor was identified by flow cytometry assay. **e** Cell–matrix adhesion assay was used to determine the adhesion ability of HeLa and C-33A cells transfected with si-NC, si-circ_0084927, miR-1179 inhibitor or si-circ_0084927 plus miR-1179 inhibitor. **f** The apoptosis of HeLa and C-33A cells transfected with si-NC, si-circ_0084927, miR-1179 inhibitor or si-circ_0084927 plus miR-1179 inhibitor was determined by the caspase 3 activation experiment. **a**–**f** The data were presented in the form of mean ± SD of three experiments. *P < 0.05, **P < 0.01, compared with the CON (blank control) group; ^#^P < 0.05, ^##^P < 0.01, compared with the si-circ (circ_0084927 siRNA) group

To investigate what role circ_0084927 plays through sponging miR-1179 in CC, we transfected circ_0084927 siRNA (si-circ_0084927), miR-1179 inhibitor or si-circ_0084927 plus miR-1179 inhibitor into HeLa and C-33A cells. We found that si-circ_0084927 decreased and increased circ_0084927 level and miR-1179 level, respectively. Meanwhile, miR-1179 inhibitor reduced miR-1179 but had no effect on circ_0084927 expression compared to the control group. In addition, si-circ_0084927 seemed to compromise the effects of the miR-1179 inhibitor on miR-1197 expression, while miR-1179 inhibitor did not in turn affect the circ_0084927 expression (Fig. 4a). The results above indicated that HeLa and C-33A cells were successfully transfected. The results of subsequent CCK-8 and BrdU incorporation assays on the successfully transfected HeLa and C-33A cells indicated that the transfection of si-circ_0084927 declined cell proliferation, while the transfection of miR-1179 inhibitor stimulated cell growth. The proliferation of the HeLa and C-33A cells co-transfected with si-circ_0084927 and miR-1179 inhibitor nonetheless had no significant change compared with the control group. This result implied that their effects could be antagonized (Fig. 4b, c).

Furthermore, flow cytometry was employed to assess cell cycle progression. Results confirmed that si-circ_0084927 significantly increased the proportion of HeLa and C-33A cells in S phase (by approximately 25% in Hela cell line and 50% in C-33A cell line) but decreased the proportion in G2/M phase (by approximately 40% in Hela cell line and 40% in C-33A cell line). In contrast, miR-1179 inhibitor reduced the proportion of cells in S phase by approximately 30% in both cell lines, which were recovered by si-circ_0084927 (Fig. 4d). Cell adhesion experiments also showed that compared to the control group, transfecting with si-circ_0084927 reduced the cell’s adhesion ability by a third; however, transfecting with miR-1179 inhibitor improved the cell’s adhesion ability by approximately 25% in both cell lines. In short, miR-1179 inhibitor transfection could restore the decrease in cell adhesion ability caused by si-circ_0084927 (Fig. 4e).

Caspase 3 is usually activated during apoptosis, irrespective of the specific death-initiating stimulus [42, 43]. This activation could reflect the cell apoptosis level, however. The results of the caspase 3 activation assay revealed that the apoptosis of HeLa and C-33A cells was significantly facilitated by circ_0084927 silence (increased by over fivefold), and repressed by miR-1179 inhibition (decreased by up to 50%). Also noted was that miR-1179 inhibition comprised the increased apoptosis caused by si-circ_0084927 (Fig. 4f).

### miR-1179 directly targeted CDK2 mRNA by binding to its 3′UTR

**Fig. 5:**
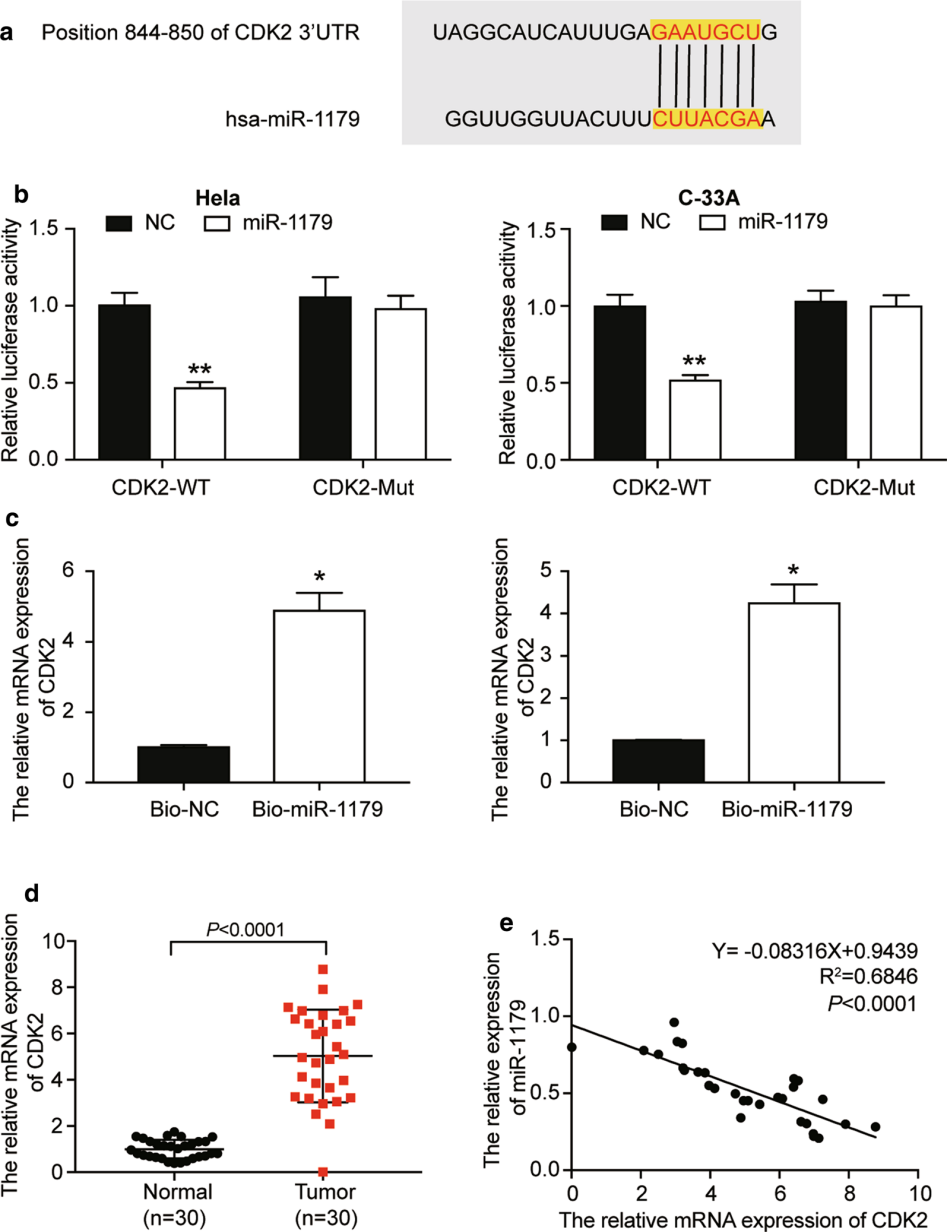
miR-1179 directly inhibited CDK2 mRNA expression by binding to its 3′UTR. **a** The potential binding site between miR-1179 and CDK2 was predicted by TargetScan Human 7.2. **b** The potential binding between miR-1179 and the 3′UTR of CDK2 gene was validated by the luciferase reporter gene assay. CDK2 mutant or CDK2 wild-type plasmids containing the fluorescence group and miR-1179 were co-transfected into HeLa and C-33A cells. **P < 0.01, compared with the NC group. NC = negative control. **c** RNA pull-down assay was used to validate the interaction between CDK2 mRNA and miR-1179. Bio = biotin-labelled. *P < 0.05, compared with the bio-NC group. **d** CDK2 expression in CC tissues and normal tissues was detected by qRT-PCR. **e** The correlation between miR-1179 and CDK2 expression was identified by spearman correlation analysis (**b**–**e**). In the three experiments, data were formatted in the form of mean ± SD

It’s predicted that miR-1179 paired with the 205–211 position of the 3′UTR of the CDK2 mRNA (Fig. 5a). The detection of the luciferase intensities demonstrated that introducing miR-1179 mimics decreased the fluorescence intensity of cells transfected with wild-type CDK2 3′UTR plasmids even though it did not affect the cells transfected with CDK2 3′UTR mutant (Fig. 5b). RNA pull-down experiment results also showed that miR-1179 interacted with CDK2 mRNA (Fig. 5c). After analyzing the CDK2 mRNA level in the obtained tissue samples, it was found that CDK2 was up-regulated in CC tissues (Fig. 5d). Findings also indicated that miR-1179 was negatively correlated with CDK2 expression (Fig. 5e).

### circ_0084927 promoted cervical carcinogenesis by sponging miR-1179 that suppressed CDK2

**Fig. 6:**
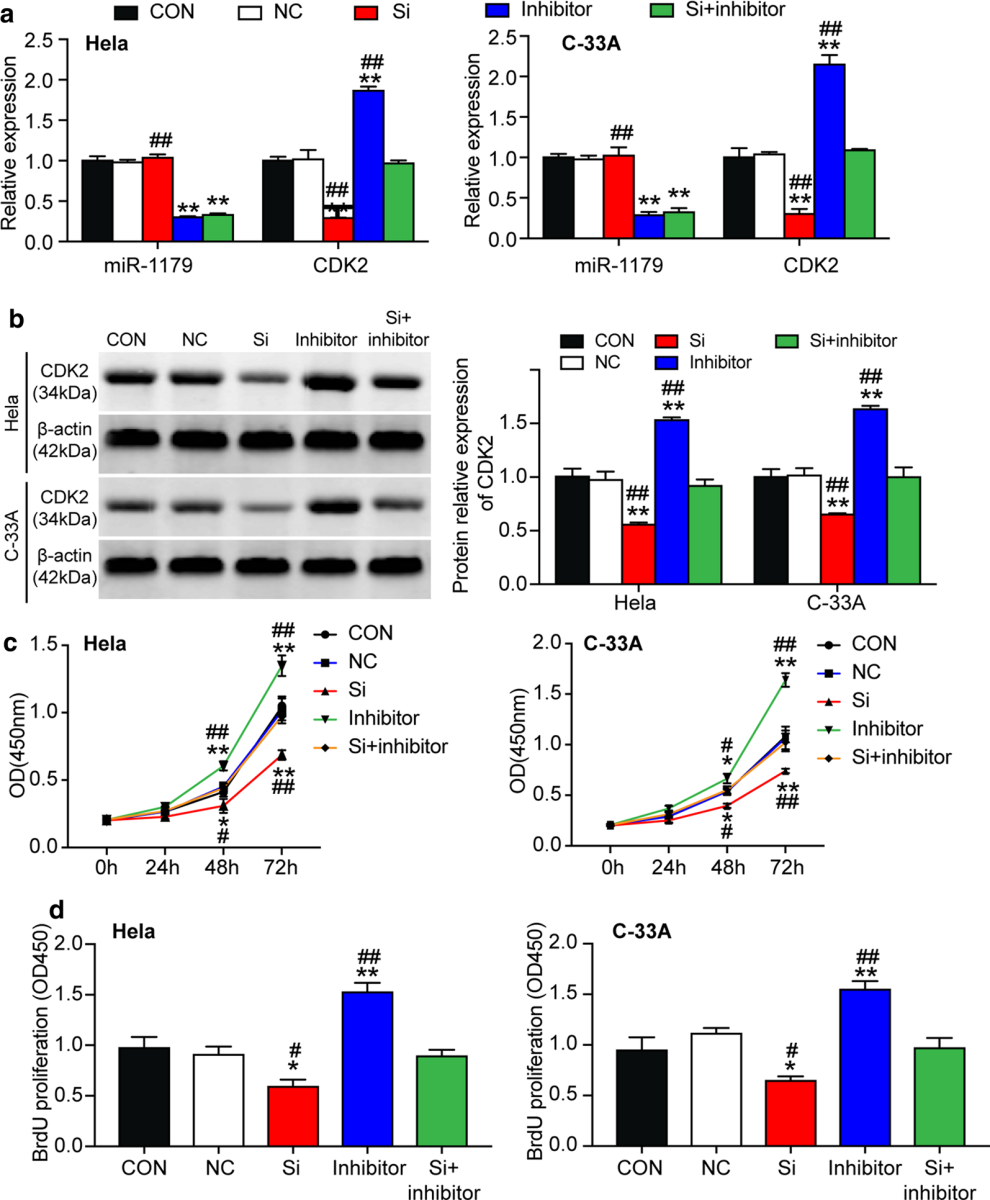
miR-1179 impaired cervical cancer cell proliferation by suppressing CDK2. **a** The transfection efficiency of HeLa and C-33A cells was measured by qRT-PCR. **b** Western blotting of CDK2 protein in transfected HeLa and C-33A cells was used to demonstrate transfection efficiency at the protein level. **c** CCK-8 assay was used to determine the proliferation of transfected HeLa and C-33A cells. **d** The proliferation of transfected HeLa and C-33A cells was determined by BrdU assay. **a**–**d** CON: blank control; NC: negative control; Si: CDK2 siRNA; inhibitor: miR-1179 inhibitor. NC, CDK2 siRNA, miR-1179 inhibitor or CDK2 siRNA plus miR-1179 inhibitor were transfected into HeLa and C-33A cells. The data of the three experiments were displayed in the form of mean ± SD. *P < 0.05, **P < 0.01, compared with the CON group, and ^#^P < 0.05, ^##^P < 0.01, compared with the Si group

To identify the effect of CDK2 expression in CC, we transfected CDK2 siRNA (si-CDK2) or miR-1179 inhibitor into HeLa and C-33A cells. Also, miR-1179 inhibitor and si-CDK2 were co-transfected into HeLa and C-33A cells to neutralize each other’s effects on CDK2 expression. qRT-PCR analysis (results in Fig. 6a) of transfected cells showed that compared with the control group, si-CDK2 transfection reduced CDK2 mRNA, while the transfection of miR-1179 inhibitor increased it. The reduction of CDK2 mRNA caused by si-CDK2 was restored by miR-1179 inhibitor, thus indicating that the cells were transfected successfully. The efficiency of si-CDK2 was approximately 70%. Western blotting analysis of CDK2 also demonstrated successful transfection at the protein level (Fig. 6b).

**Fig. 7:**
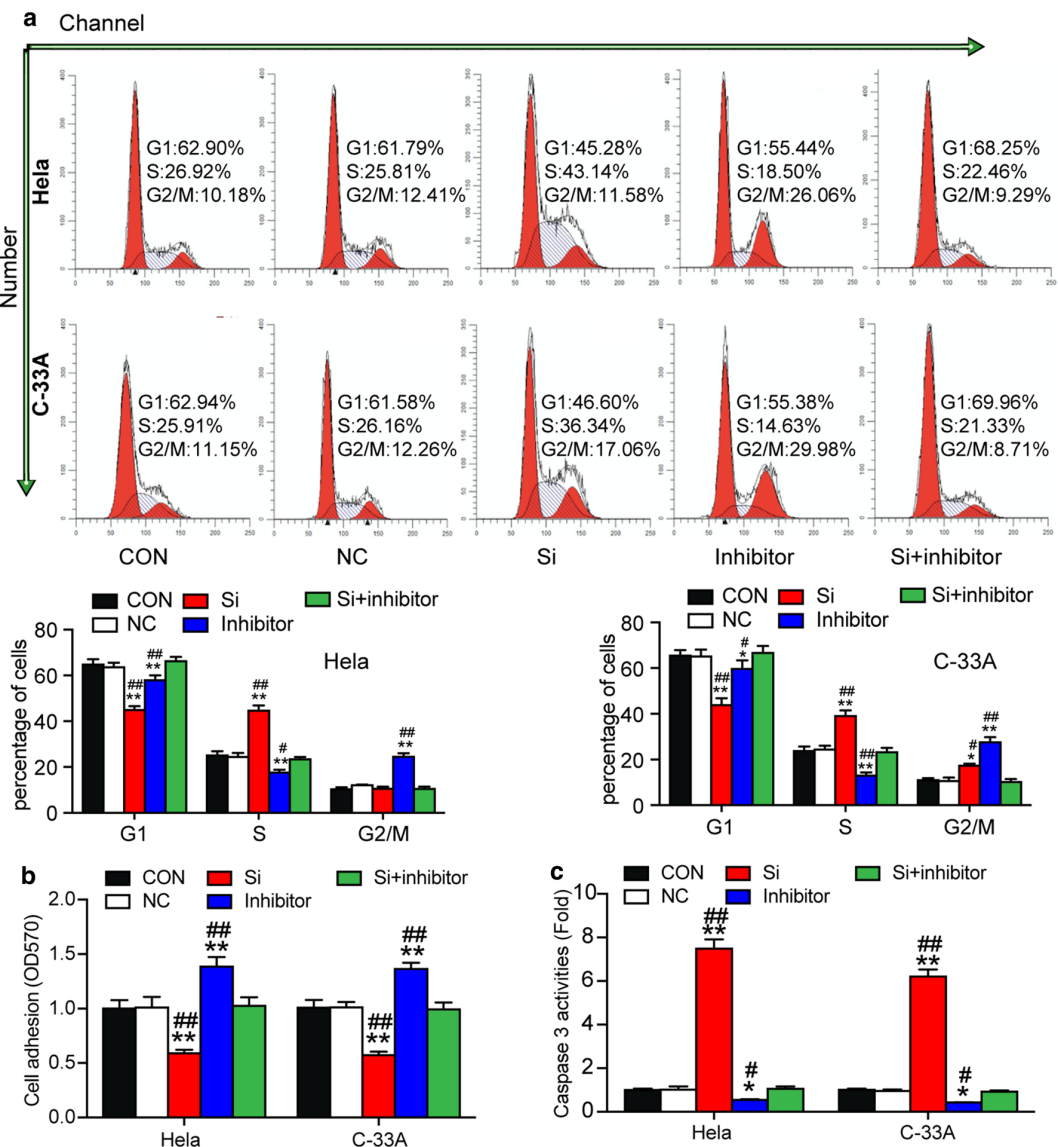
miR-1179 impaired cervical cancer cell cycle progression and adhesion and enhanced the apoptosis by suppressing CDK2. **a** The cell cycle progression of transfected HeLa and C-33A cells was identified by flow cytometry assay. **b** Cell–matrix adhesion assay was used to determine the adhesion ability of transfected HeLa and C-33A cells. **c** The apoptosis of the transfected HeLa and C-33A cells was determined by Caspase 3 activation experiment. **a**–**c** CON: blank control; NC: negative control; Si: CDK2 siRNA; inhibitor: miR-1179 inhibitor. NC, CDK2 siRNA, miR-1179 inhibitor or CDK2 siRNA plus miR-1179 inhibitor were transfected into HeLa and C-33A cells. The data of the three experiments were arranged in the form of mean ± SD. *P < 0.05, **P < 0.01, compared with the CON group, and ^#^P < 0.05, ^##^P < 0.01, compared with the Si group

We explored the cellular phenotypes of the HeLa and C-33A cells transfected successfully. The results of the CCK-8 assay indicated that silencing CDK2 attenuated HeLa and C-33A cell proliferation, while miR-1179 down-regulation offset the inhibitory effect caused by CDK2 silence at 48 h and 72 h (Fig. 6c). The BrdU experiment also showed the same cell proliferation results: CDK2 silencing inhibited cell proliferation (Fig. 6d). While silencing CDK2 significantly augmented the proportion of S-phase cells compared to the control group, it diminished the proportion at the G1 phase. Besides, the growth in the proportion of S-phase cells caused by CDK2 silencing was attenuated by the down-regulation of miR-1179 (Fig. 7a). In short, CDK2 silencing significantly reduced HeLa and C-33A cell adhesion, and this reduction was restored by simultaneous down-regulation of miR-1179 (Fig. 7b). Finally, in caspase 3 activation experiments, CDK2 silencing increased HeLa and C-33A cell apoptosis, which was compromised by miR-1179 down-regulation (Fig. 7c).

## Discussion

Many studies have reported the up-regulation and miRNA-sponging roles of circRNAs in CC. For instance, circ_101996 was overexpressed in cervical cancerous tissues [44]. One study showed that circ_0084927 was significantly up-regulated in malignant pleural effusion (MPE) of lung cancer [45]. We reported in this research the significant up-regulation of circ_0084927 in CC tissues and cell lines, and we hypothesized that the upregulated circ_0084927 might facilitate CC progression. Most importantly, we proved that circ_0084927 silencing inhibited carcinogenesis by repressing the proliferation and adhesion of CC cells, fortifying apoptosis, and leading to cell cycle arrest. Overall, circ_0084927 promoted CC occurrence through the regulatory network of circ_0084927/miR-1179/CDK2.

Many up-regulated circRNAs were identified to exert critical tumor-promoting functions by sponging its downstream miRNAs. For instance, circ_0023404 and hsa_circ_CLK3 stimulated the aggressiveness of CC cells by acting as a miRNA sponge, thereby facilitating cancer occurrence and metastasis [46, 47]. What’s more, circ_0000515 served as a miR-326 sponge, thus promoting the progression of CC [18, 48]. According to a recent study, up-regulated circ_0000388 dramatically stimulated CC aggression by sponging miR-337-3p [49]. In another research, circ_0075341 was identified as a sponge of miR-149-5p to promote the malignant phenotypes of CC cells [19]. Similarly, circ_0060467 stimulated aggressiveness of CC by sponging miR-361-3p [50].

We herein identified a potential downstream miRNA of circ_0084927, miR-1179, which has not been comprehensively studied in several cancers except CC. We found that circ_0084927 promoted CC malignant phenotypes by sponging miR-1197. This result suggested that circ_0084927 exerted its tumor-promoting functions by suppressing miR-1197, which was once described as a tumor suppressor that impaired the malignant progression of gastric cancer by inhibiting proliferation and invasion [30]. Another study showed that increased miR-1179 significantly inhibited the aggressiveness of breast cancer cells while weakening the cancer metastasis [33]. Moreover, miR-1179 functioned like a tumor suppressor: It inhibited the malignant proliferation and cell cycle progression of glioblastoma multiforme cells [29]. These results suggested that miR-1179 could be a potential tumor suppressor in diverse cancers. However, no previous studies, which showed that miR-1179 played a tumor suppressor role in CC, have been reported.

We herein supplemented the results in CC and proposed that miR-1179 played an anti-oncogenic role in CC. It is crucial to note that miR-1179 has been reported to interact with other circRNAs, thus affecting human cancer cell phenotypes. For instance, a previous study published that the tumor suppressor function of miR-1179 was confiscated by circ_0000735 during the development of non-small cell lung cancer [51]. A similar experiment confirmed that the inhibitory effect of miR-1179 on thyroid cancer was sponged by circ_0039411 during the pathological process [52]. Another study pointed out that circ_0003645 improved cell aggressiveness through sponging miR-1179 [53]. As a cancer-promoting factor, circ_0025033 promoted the progression and tumor growth of papillary thyroid carcinoma by sponging miR-1179 [54]. These studies supported the claims that miR-1179 could exert tumor suppressor functions by sponging with circRNAs. Apart from validating the regulatory relationship between circ_0084927 and miR-1179, our findings unraveled the tumor-promoting effect of circ_0084927 and expanded the regulatory networking involving miR-1179 in CC. Most importantly, we identified a novel regulatory interactome that might contribute to the understanding of CC pathogenesis.

Regarding the downstream effector of miR-1179, CDK2, several studies have reported that CDK2 is the downstream effector of miRNAs in various cancers. This means that it affected cancer cell malignancy, especially cell-cycle progression. For instance, silencing CDK2 attenuated aerobic glycolytic cell metabolism in cells, thereby inhibiting the malignant characterization of gastric cancer cells [55]. CDK2 was also up-regulated in many cancers as a cell cycle-dependent kinase that contributed to cell cycle progression and DNA damage responses [56]. This up-regulation of CDK2 provided new immune targets for therapy on multiple cancers.

The study of CDK2 inhibitors also provided new prospects for cancer treatment [57, 58]. Our results, for example, showed that the inhibition of CDK2 significantly suppressed CC cell growth and cell cycle progression as well as cell–matrix adhesion. Consistent with previous studies, our findings indicated that CDK2 could be a valuable therapeutic target for CC treatment. In terms of the interaction of miRNAs and circRNAs that are upstream of CDK2, it was reported that by downregulating miR-3619-5p, CDK2 exerted a crucial role in promoting the proliferation, migration and invasion of bladder carcinoma cells [59]. By regulating its upstream circ_0078710/miR-31, CDK2 stimulated the malignant phenotypes of hepatocellular carcinoma cells [60]. CDK2 was noted in another research to form a complex substance with circ-Foxo3; this substance was abnormally expressed in cancer tissues in terms of participating in cell cycle regulation [61]. The evidence above indicated that CDK2 could be downstream effectors of circRNAs and miRNAs.

In our study, we reported a novel upstream regulator of CDK2, circ_0084927/miR-1179, in CC. We also found that miR-1179 inhibited the malignant phenotypes, including cell cycle progression of CC cells, by directly targeting CDK2. This regulation, however, could be reversed by circ_0084927 because it could sponge miR-1179 to release CDK2. To better clarify the pathogenesis of CC, the results of the in vitro experiments performed in this study require the validation of animal models. Even though our study did not further investigate the molecular receptors downstream of the CDK2 in CC, the accumulation of clinical samples is required to expand the sample size and ensure that the results are compelling and convincing.

## Conclusion

In summary, this study indicated that circ_0084927 stimulated CC by sponging miR-1179, which negatively targeted CDK2. Our results revealed the existence of a complex circ_0084927/miR-1179/CDK2 axis in cervical carcinogenesis. In terms of the search for CC therapy, our study highlighted the possibility of circ_0084927 as a candidate target.

## Supplementary information

**Additional file 1: Figure S1.** The representative histopathological examination images from 33 cervical cancer patients by H&E staining.

**Additional file 2: Figure S2.** The Metasacape.org analysis results of the differentially expressed genes (DEGs) of GSE63514 data series. (A) A bar graph showing the enriched terms across the 904 input DEGs list. The bar was colored by P values. (B) The MCODE components identified in the input DEGs list. The criteria for DEGs were adj. P < 0.01 and log|FC| ≥ 2.

**Additional file 3: Figure S3.** GSEA analysis was done on the DEGs of the GSE63514 data series, and it was found that cell cycle KEGG pathway was significantly upregulated in cervical cancer.

**Additional file 4: Figure S4.** GSEA analysis was carried on the DEGs of the GSE63514 data series, and it was found that the regulation of cell cycle phase transition GO biological process was significantly upregulated in cervical cancer.

**Additional file 5: Figure S5.** GSEA analysis was performed on the DEGs of the GSE63514 data series, and findings revealed that the cell-cycle checkpoint GO biological process was significantly upregulated in cervical cancer.

## Data Availability

The data used in the current study are available from the corresponding author on reasonable request.

## Abbreviations

miRNAs: MicroRNAs
PBS: Phosphate buffered solution
BrdU: 5-Bromo-2-deoxyuridine
CC: Cervical cancer
qRT-PCR: Quantitative real-time PCR
