# Supplementary figures and images for: circ_0084927 promotes cervical carcinogenesis by sponging miR-1179 that suppresses CDK2, a cell cycle-related gene
Source: Cancer Cell Int. 2020 Jul 21;20:333. doi: 10.1186/s12935-020-01417-2 (PMC7372805; doi:10.1186/s12935-020-01417-2)

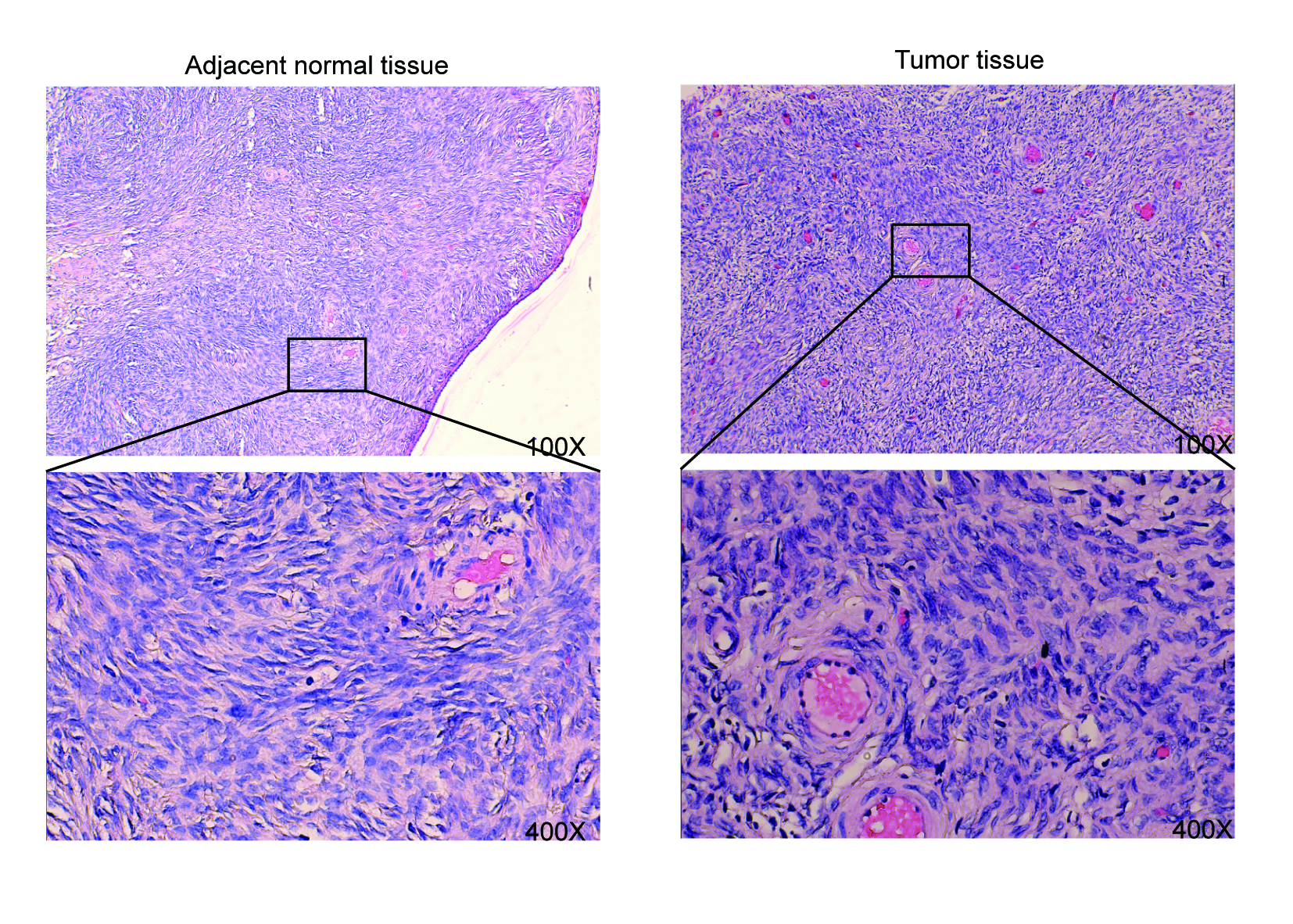

Supplement: Supplementary file 1 — Additional file 1: Figure S1. The representative histopathological examination images from 33 cervical cancer patients by H&E staining. [file 12935_2020_1417_MOESM1_ESM.tif]

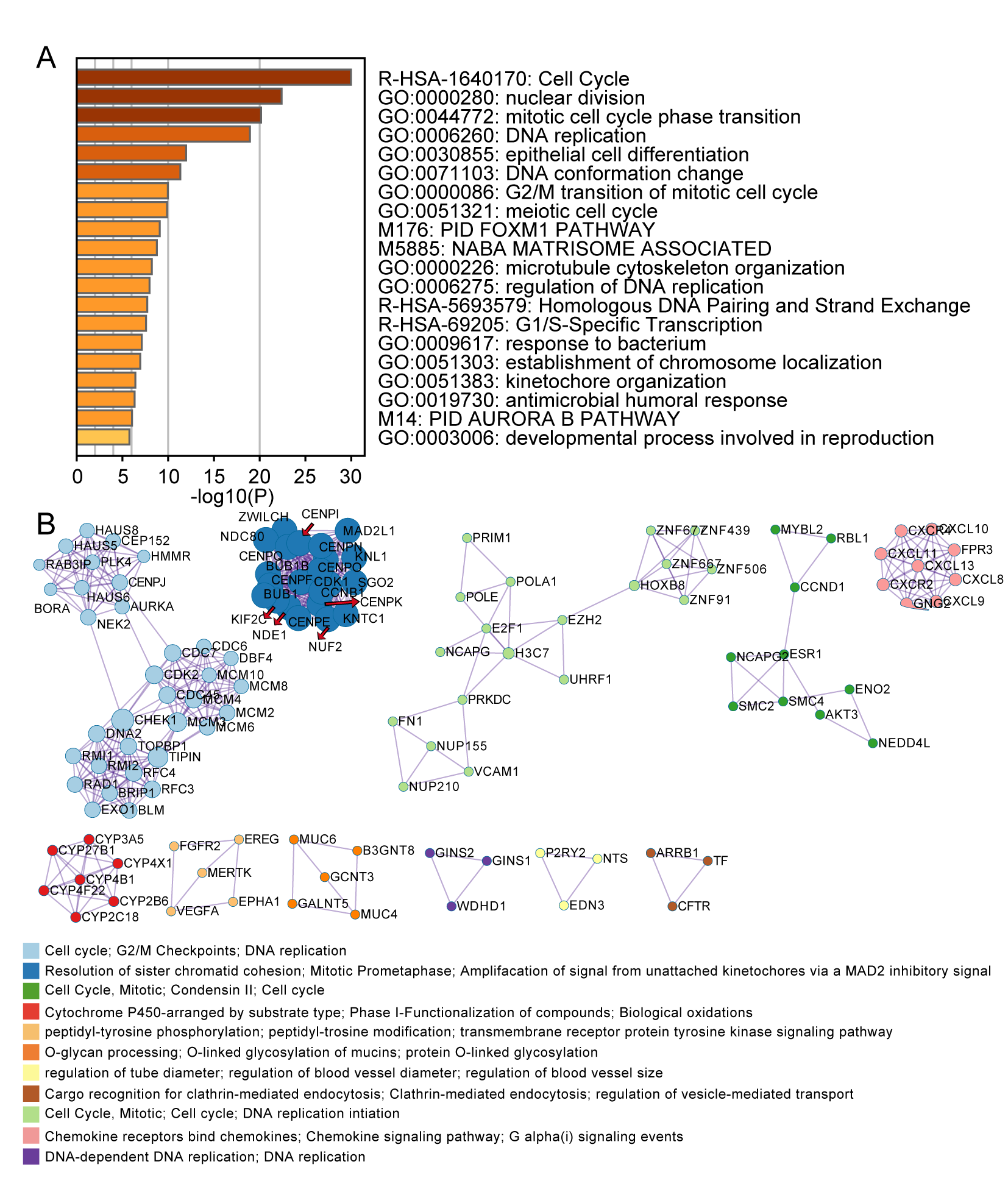

Supplement: Supplementary file 2 — Additional file 2: Figure S2. The Metasacape.org analysis results of the differentially expressed genes (DEGs) of GSE63514 data series. (A) A bar graph showing the enriched terms across the 904 input DEGs list. The bar was colored by P values. (B) The MCODE components identified in the input DEGs list. The criteria for DEGs were adj. P < 0.01 and log|FC| ≥ 2. [file 12935_2020_1417_MOESM2_ESM.tif]

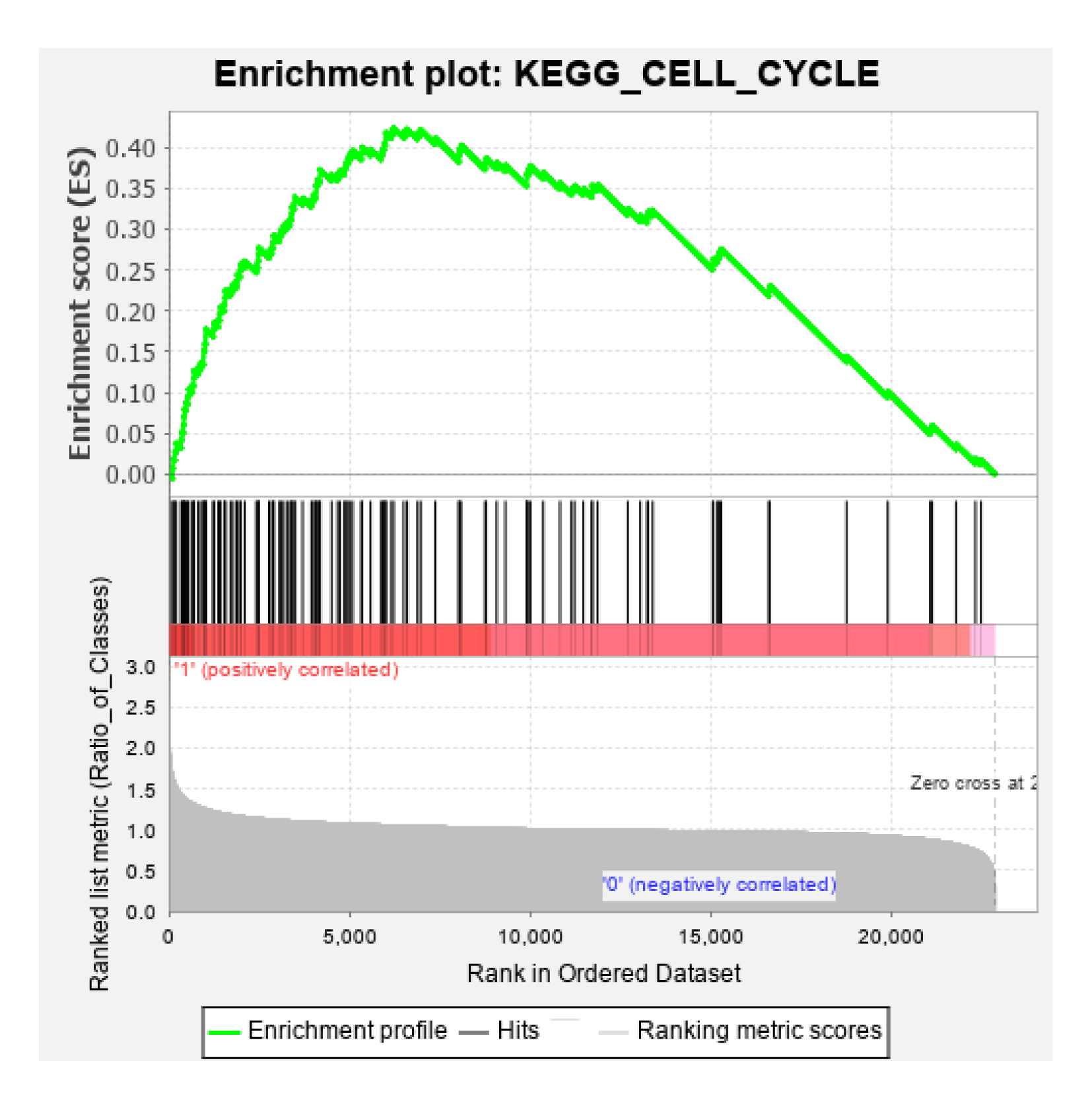

Supplement: Supplementary file 3 — Additional file 3: Figure S3. GSEA analysis was done on the DEGs of the GSE63514 data series, and it was found that cell cycle KEGG pathway was significantly upregulated in cervical cancer. [file 12935_2020_1417_MOESM3_ESM.tif]

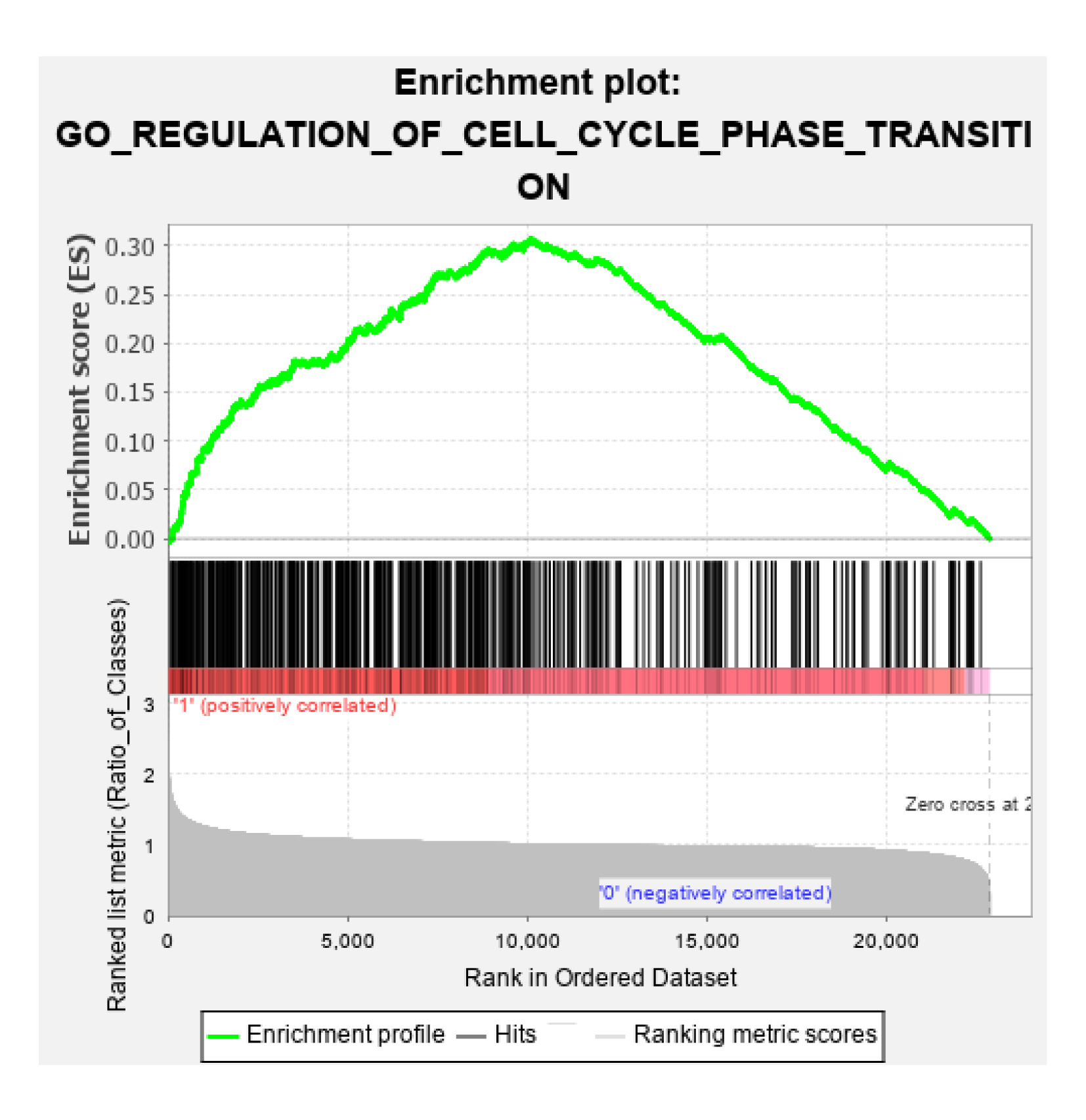

Supplement: Supplementary file 4 — Additional file 4: Figure S4. GSEA analysis was carried on the DEGs of the GSE63514 data series, and it was found that the regulation of cell cycle phase transition GO biological process was significantly upregulated in cervical cancer. [file 12935_2020_1417_MOESM4_ESM.tif]

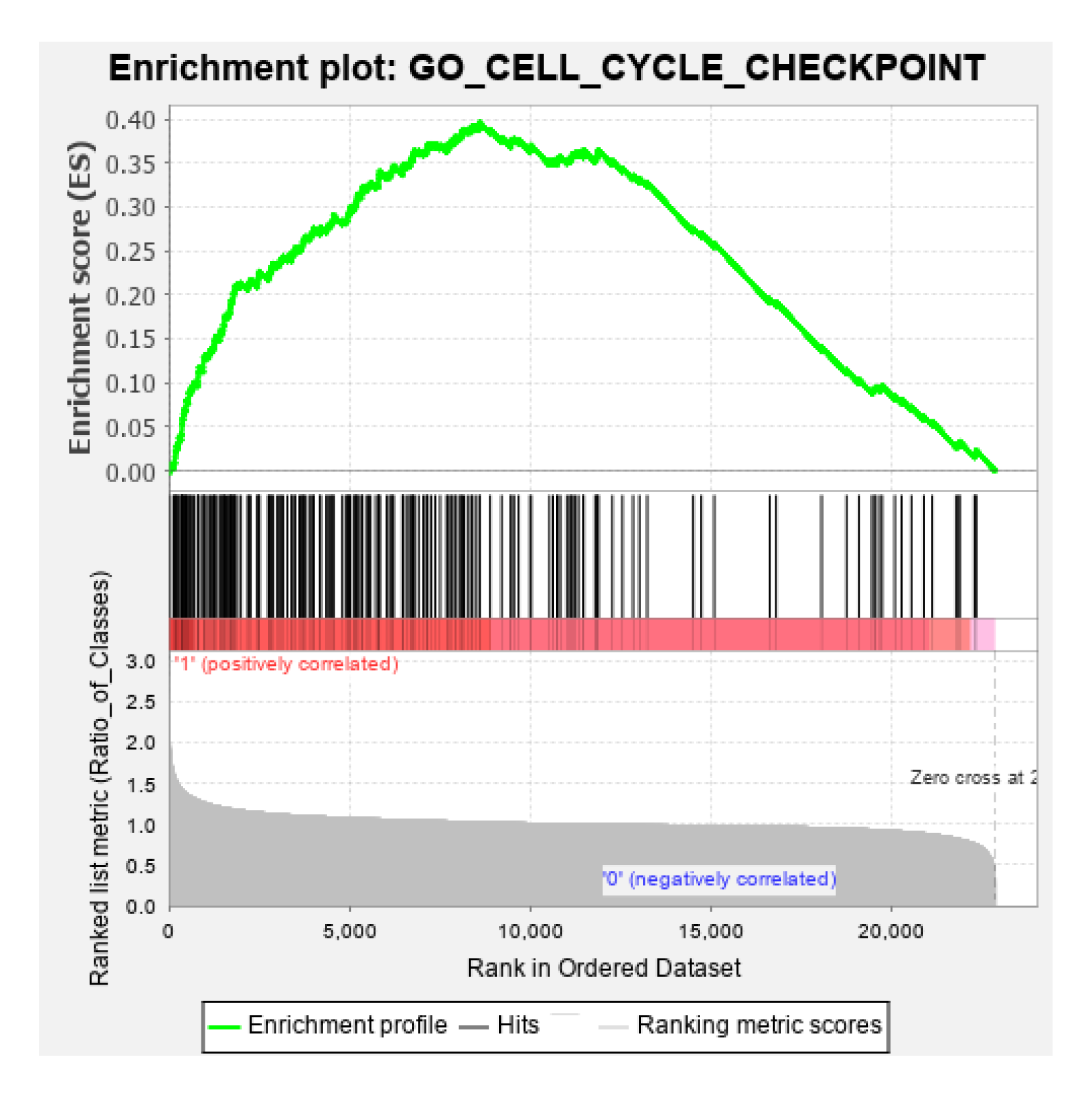

Supplement: Supplementary file 5 — Additional file 5: Figure S5. GSEA analysis was performed on the DEGs of the GSE63514 data series, and findings revealed that the cell-cycle checkpoint GO biological process was significantly upregulated in cervical cancer. [file 12935_2020_1417_MOESM5_ESM.tif]
